# Strengthening from Cu Addition in 0.2C-(1-2)Mn Steels during Tempering

**DOI:** 10.3390/ma12020247

**Published:** 2019-01-13

**Authors:** Jaromir Dlouhy, Pavel Podany, Jan Dzugan

**Affiliations:** COMTES FHT a.s., Prumyslova 995, 334 41 Dobrany, Czech Republic; pavel.podany@comtesfht.cz (P.P.); jan.dzugan@comtesfht.cz (J.D.)

**Keywords:** low carbon steel, copper precipitation, manganese, precipitation strengthening

## Abstract

The strengthening effects of Cu and Mn were studied in steels, which contained 0.2% C and were micro-alloyed with B and Ti. The experimental steels were austenitized and quenched in order to take Mn and Cu into solid solution. The subsequent tempering of martensitic structures resulted in higher strengths in the materials alloyed with Cu than in the steel without Cu addition. Tensile testing and metallographic analyses were performed. The kinetics and magnitude of precipitation strengthening were measured for different tempering temperatures and times. Presumed synergistic effects between Cu precipitation strengthening and higher levels of Mn were observed.

## 1. Introduction

Copper precipitation has been thoroughly studied over time. It causes precipitation strengthening of ferrite. Cu has low solubility in the ferrite matrix at room temperature; and precipitates out from supersaturated solution as globular precipitates. Cu precipitation occurs when the Cu content is higher than 0.5%. Studies of microstructure revealed that Cu precipitates start to grow as particles with bcc structure coherent with the matrix [1,2,3]. The surrounding ferritic matrix keeps the non-equilibrium bcc lattice of the Cu particle until the precipitate reaches a certain size. The precipitate then undergoes phase transformation into a 9R orthorhombic lattice phase, and finally adopts the equilibrium Cu fcc crystal lattice and loses coherency with the ferrite. 

Copper precipitation causes strengthening of the ferrite matrix. Yield stress (YS) of the ferrite rises by up to 200 MPa per 1 wt.% of Cu [4]. The maximum strengthening was ascribed to the phase of coherent precipitates because of the stress fields in the ferritic matrix surrounding the precipitate [5,6]. However, some studies [7,8] imply that there is no abrupt change in the amount of precipitation strengthening with the precipitate’s transition from the coherent to the incoherent form and that the strengthening mechanism depends mainly on the size of and the average distance between the precipitates.

This article is devoted to quenching and tempering of steels with 0.2% C and different contents of Cu and Mn (all concentrations in this article are presented in wt.%). Their alloying concepts include additions of B and Ti for increased hardenability and possible synergies with Cu precipitation [9,10,11,12,13]. The aim of the experimental program was to determine the effects of Cu and Mn on mechanical properties and to reveal possible synergy or antagonism between these elements in terms of strengthening.

## 2. Materials and Methods

Five experimental heats were manufactured and cast. Their chemical compositions varied in the contents of Cu and Mn. The concentrations of the other chemical elements were reproduced as precisely as possible in all five melts. The chemical compositions are given in Table 1. They were measured with the Q4 TASMAN optical emission spectrometer (Bruker Elemental GmbH, Kalkar, Germany), which was used for the solid elements, and with the G8 GALILEO gas fusion analyzer spectrometer (Bruker Elemental GmbH, Kalkar, Germany), which was employed to determine the nitrogen content. Titanium was added as a binding agent for nitrogen. Free nitrogen could form the boron nitride and thus remove boron from the solid solution and suppress its effect. Ti was added in an amount, which exceeded the assumed nitrogen content by a factor of 4, when expressed in wt.%. The heats were labelled with respect to their Cu and Mn contents, which were the only elements whose levels varied significantly among the experimental steels. The reference melt without any intentionally added Cu is designated 0Cu, although there is Cu present in this material. The copper content in the 0Cu heat is in the range ordinarily found in steel and is too low to induce Cu precipitation.

The heats were cast in an induction vacuum furnace (První železářská společnost Kladno, s.r.o., Kladno, Czech Republic) in an argon atmosphere; the batch size was 500 kg. Ingots of round cross sections were hot forged in a hydraulic press (První železářská společnost Kladno, s.r.o., Kladno, Czech Republic) into billets 300 × 80 mm^2^ in cross section. These billets were milled to remove the casting skin and hot rolled into sheets 300 mm wide and 5 mm thick. The sheets were then descaled by sandblasting. Cold rolling of the descaled sheets provided them with the final flat surface and a uniform thickness of 3.2 mm. Samples with dimensions of 250 × 75 mm^2^ were cut from these sheets. Their longer sides were parallel to the rolling direction.

Thermal treatment consisted of quenching and tempering. The quenching temperature was 870 °C, with a soak time of 30 min. The samples were covered with protective coating against decarburization, heated in an air furnace (BVD pece spol. s.r.o., Podlesí, Czech Republic) and quenched into a water bath.

Tempering took place in the air furnace as well. Tempering temperatures were 300, 400 and 500 °C; with tempering times of 15, 30, 60 and 120 min. 

Mechanical properties were determined by tensile testing and hardness measurement. Three tensile test specimens were machined from each sample. These flat specimens had the thickness of the original sheet (from 3.1 to 3.3 mm), a gauge length of 30 mm and a width of 10 mm. Quasistatic tensile tests were performed according to the EN ISO 6892-1 standard [14], with a deformation rate of 1 mm/min, using a Zwick Z250 testing machine with a 250 kN capacity (ZwickRoell GmbH & Co. KG, Ulm, Germany). Ultimate tensile strength (UTS), yield stress (YS), homogeneous plastic elongation (Ag) and total plastic elongation (elongation A_5_) were determined. YS was determined as the proof stress at 0.2% offset.

Metallographic analysis was performed using light microscopy (NIKON ECLIPSE MA200; NIKON, Tokyo, Japan), scanning electron microscopy (SEM) and scanning transmission electron microscopy (STEM). The SEM instrument was JEOL IT 500 HR (JEOL, Tokyo, Japan) and the STEM instrument was SEM/FIB (scanning electron microscopy/focused ion beam) microscope Carl Zeiss CrossBeamAuriga (Carl Zeiss AG, Oberkochen, Germany). Sections for microscopic observation were prepared in the longitudinal direction of specimens by mechanical grinding and polishing. The microstructure was revealed by etching with 3% Nital. A thin lamella for STEM observation was extracted from the surface of one metallographic section by focused ion beam milling. Gallium cations were used for the milling process.

The prior austenite grain (PAG) size was measured using a light microscope (Nikon Eclipse MA200, Nikon, Yokohama, Japan). Metallographic sections were etched with a saturated aqueous solution of picric acid with an addition of surfactant at 80 °C. The PAG size was determined by measuring the average grain intercept length according to the EN ISO 643 standard [15] at magnification 500×.

## 3. Results

### 3.1. Initial State

Sheets were subjected to hot- and cold rolling prior to the heat treatment. Their final thickness was 3.2 mm. Microstructure was multiphase, composed of ferrite and the other structural constituent was mixture of bainite and martensite. There were visible particles of TiN in the structure. Formation of TiN binds nitrogen and prevents formation of boron nitride [16,17]. TiN particles are thermally stable to temperatures exceeding 1300 °C [18,19]. It is not expected that subsequent thermal treatment with austenitization at 870 °C caused their dissolution and release of nitrogen into solid solution.

**Figure 1:**
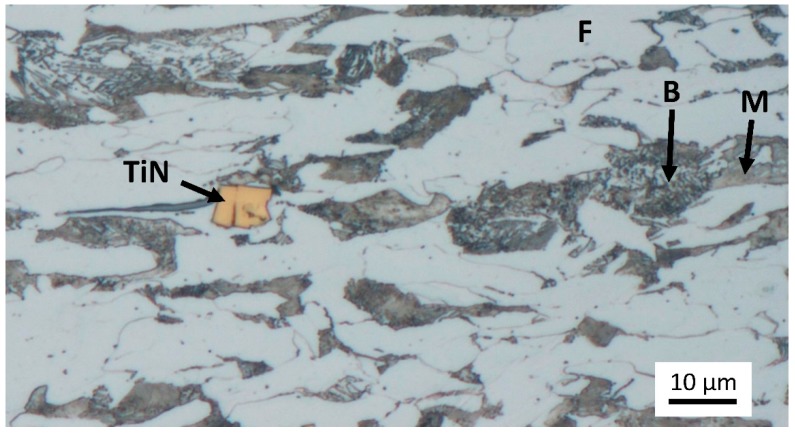
Microstructure of the 0Cu-1Mn after cold rolling to final thickness 3.2 mm. Rolling direction is horizontal in the image. Microstructure was composed of ferrite (F), bainite (B) and martensite (M). TiN particles were observed in the structure.

The TiN particles were up to 8 µm in diameter. This rather large particles can crack during the forming process, as can be seen in Figure 1. Microcracks formed in the TiN particle can act as fracture initiation when the material is stressed.

### 3.2. Quenched State

**Figure 2:**
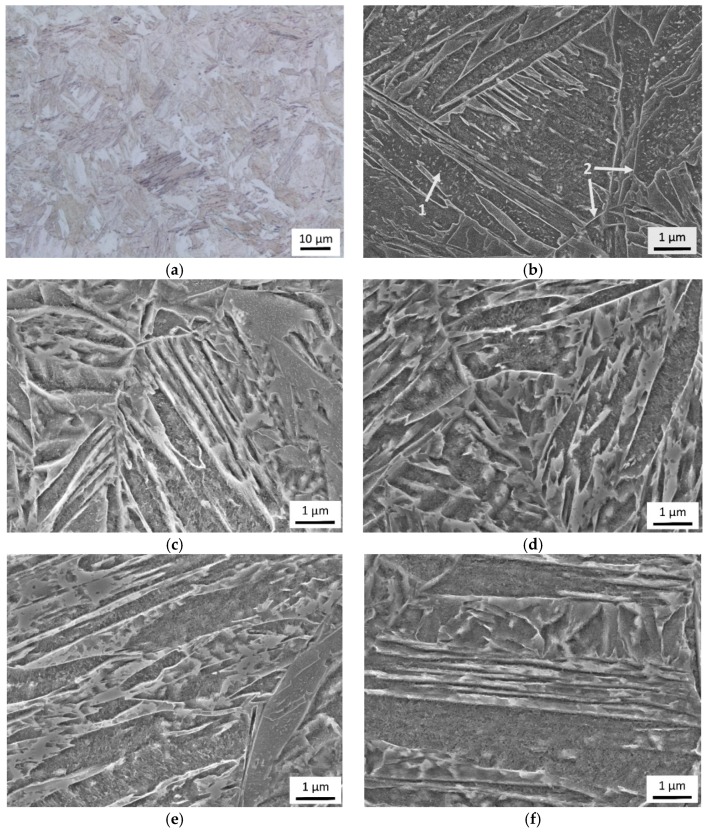
Microstructure of the materials after quenching. Martensitic microstructure of 0Cu-1Mn in: (**a**) light microscope; (**b**) SEM—martensite crystals, some show fine elongated particles (arrow 1), PAG grain boundaries (arrow 2). SEM images of martensitic microstructure in materials: (**c**) 1Cu-1Mn; (**d**) 1Cu-2Mn; (**e**) 1.5Cu-1Mn; (**f**) 1.5Cu-2Mn.

Mechanical properties of the materials in the as-quenched state are listed in Table 2. UTS and YS generally increased with the contents of Cu and Mn, whereas Ag slightly decreased. A_5_ and hardness did not exhibit any trend. The microstructures of all the materials were martensitic after quenching (Figure 2). They had the same appearance in micrographs and showed no significant variations in the PAG size (see Table 2). The martensitic structure was homogeneous and did not exhibit any banding. The rolling direction of the sheets was not distinguishable in the microstructure (Figure 2a). A detailed SEM inspection revealed individual martensite crystals in the structure.

They can be seen in Figure 2b, delineated by bright lines, as are the PAG boundaries. The morphologies and sizes of martensite crystals and the appearance of their boundaries were visually identical in all the as-quenched materials. Fine elongated particles were observed within and in between some martensite crystals (Figure 2b).

### 3.3. Tempered State

**Figure 3:**
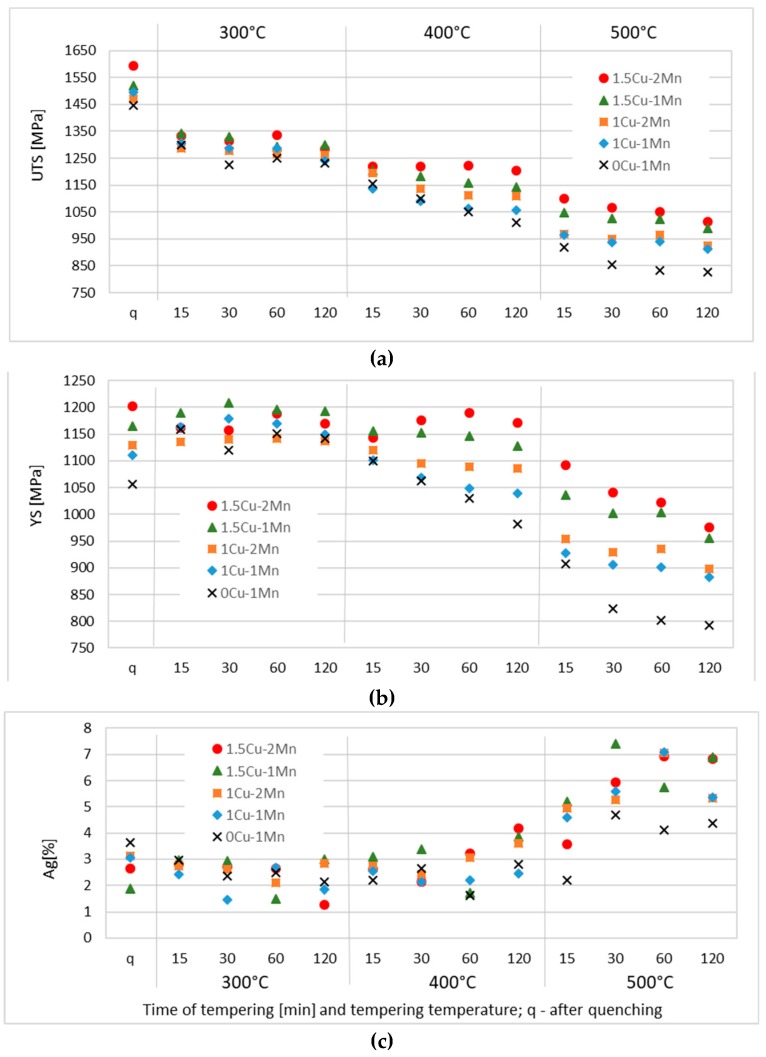
Mechanical properties of samples after quenching and tempering. (**a**) UTS ultimate tensile strength; (**b**) YS—yield stress; (**c**) Ag—uniform plastic elongation.

Results of the tensile tests are presented in Figure 3 for individual tempering temperatures and times. UTS, YS and Ag are listed. The A_5_ elongation followed exactly the trend of the homogeneous plastic elongation Ag, but its values exhibited a larger scatter. Therefore, the graph does not include the A_5_ values.

Tempering at 300 °C resulted in a sharp drop in UTS for all materials. YS, however, did not decrease at all. The difference between UTS and YS was smaller in all tempered samples than in the as-quenched ones. The differences between the corresponding values for the lowest and the highest- alloyed materials decreased from roughly 150 MPa in the as-quenched state to 75 MPa in the tempered state. No strong dependence of UTS and YS on alloying was identified, as with the dependence on the time of tempering. Ag did not increase with the intensity of tempering. In fact, it even slightly decreased on average. It is important to note that the scatter in Ag values was significant and that no definite conclusions can be drawn from them.

The tempering temperature of 400 °C caused a different effect. The dependence of YS on tempering time was significantly different for the reference material without Cu and for the heats that contained 1 and 1.5% Cu. The 0Cu-1Mn material showed a monotonous decrease in both UTS and YS with increasing tempering time. The materials with 1% Cu exhibited a similar decrease, but the its rate for UTS and YS was slower. This resulted in a broadening difference between the strengths of the reference material 0Cu and the materials with 1% Cu. In the 1.5Cu materials, UTS and YS were higher after 60-min tempering than after 15-min tempering but slightly lower after 120 min. The difference between the lowest and the highest-alloyed materials increased from roughly 75 MPa (for 15-min tempering) to 200 MPa for 60 and 120 min of tempering at 400 °C. In the graph, the heats appeared “stratified” according to their Cu and Mn contents after longer tempering times. Ag did not exhibit any significant trend. It was slightly higher after the longest, 120-min tempering cycle but the values showed a large scatter.

Tempering at 500 °C led to a monotonous decrease in UTS and YS with increasing tempering time for the lowest and the highest-alloyed materials. The difference between these two materials in their respective UTS and YS values was the largest among all the tempering regimes (up to 220 MPa). Interestingly, the materials 1Cu-1Mn, 1Cu-2Mn and 1.5Cu-1Mn exhibited a plateau or even a slight increase in UTS and YS plots between the tempering times of 30 min and 60 min. As before, the materials were ordered based on their alloying in the graph. The differences between individual heats were determined mainly by the Cu content, whereas the Mn content only had a minor influence (compare the values in Figure 3a,b for 500 °C with the lower tempering temperatures or the as-quenched state). Ag increased with the tempering time up to the 30-min regime, and then remained constant.

**Figure 4:**
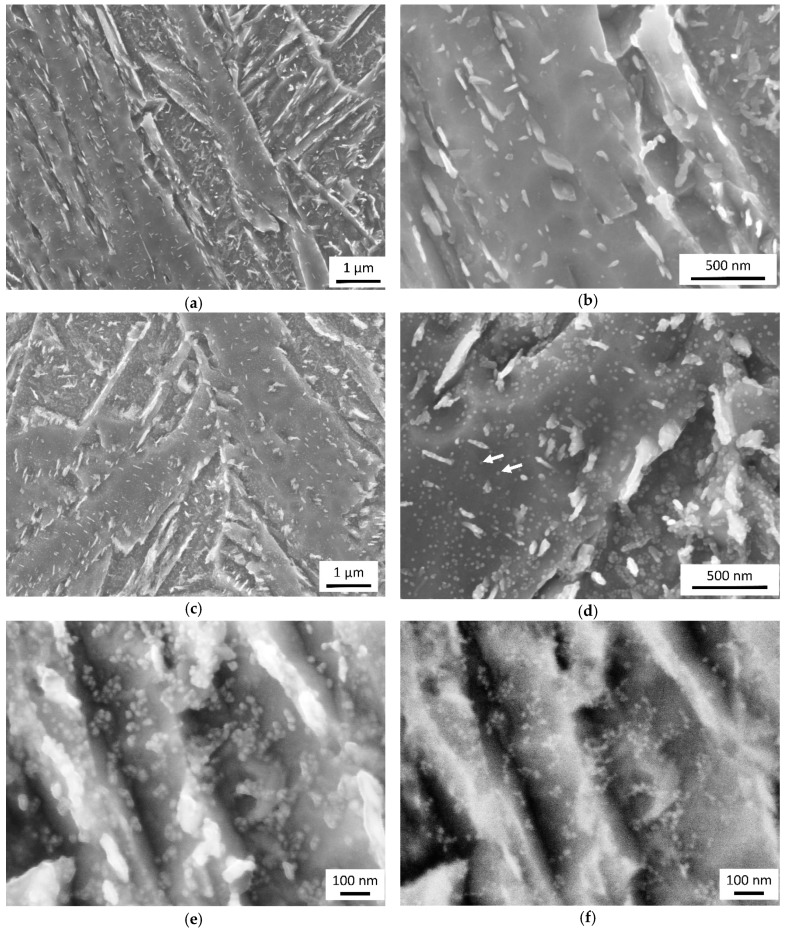
SEM micrographs of samples quenched and tempered at 400 °C for 30 min: (**a**) Material 0Cu-1Mn exhibited a structure of tempered martensite; (**b**) Detail from panel (**a**) with clearly distinguishable cementite particles; (**c**) Material 1.5Cu-2Mn exhibited a structure of tempered martensite; (**d**) Detail from the panel (**c**) with small globular particles in the ferritic matrix beside cementite particles; (**e**) particles starting to be blurry at high magnification in secondary electron image; (**f**) the same image field as panel (**e**) captured in backscattered electrons in enhanced element contrast. Small bright particles stand out from the ferritic matrix.

All the materials exhibited a microstructure of tempered martensite. The microstructures of different materials for the same tempering parameters were not distinguishable at lower magnifications (compare Figure 4a,c). The size, morphology and distribution of cementite particles were basically the same, for those within martensite crystals, as well as those in between them. Observation at higher resolutions revealed a difference between the reference material 0Cu and the materials alloyed with Cu. Figure 4b,d show details of the microstructures of the lowest and highest-alloyed materials after tempering at 400 °C for 30 min. The difference is clearly visible. Numerous globular particles, not larger than 30 nm, can be seen in material 1.5Cu-2Mn. Further increase in magnification revealed aggregation of particles released by etching (Figure 4e). Their appearance changed when captured in mode of backscattered electrons (Figure 4f). They appeared smaller in diameter and their position was more precisely located in spite of higher noise of the image.

**Figure 5:**
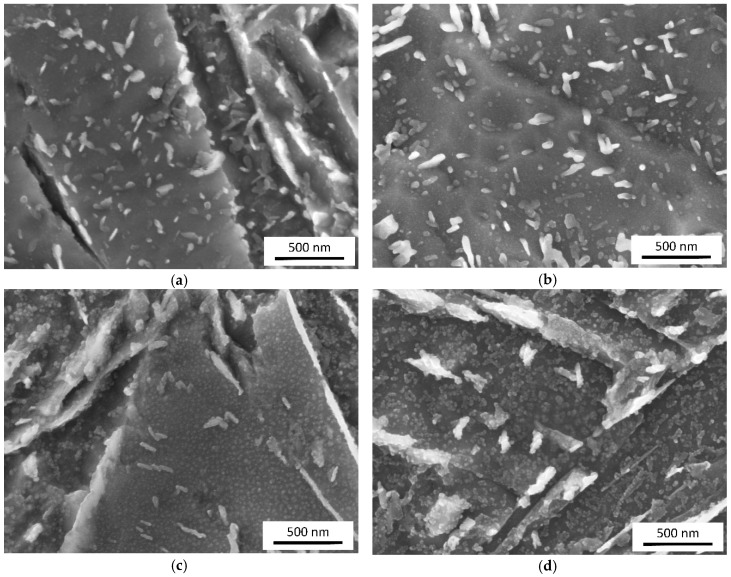
Comparison of tempered martensite microstructures in the steels with the lowest and highest alloying element content: Material 0Cu-1Mn tempered for 60 min at (**a**) 300 °C; (**b**) 500 °C; material 1.5Cu-2Mn tempered for 60 min. at (**c**) 300 °C; (**d**) 500 °C.

There were observed particles in tempered martensite for all Cu-containing materials. These particles appeared more distinctive with increased time and temperature of tempering (Figure 5). They were not observed in any sample of material 0Cu-1Mn. Therefore, it can me assumed, that the observed particles in size up to 30 nm represents Cu precipitates in ferrite matrix.

**Figure 6:**
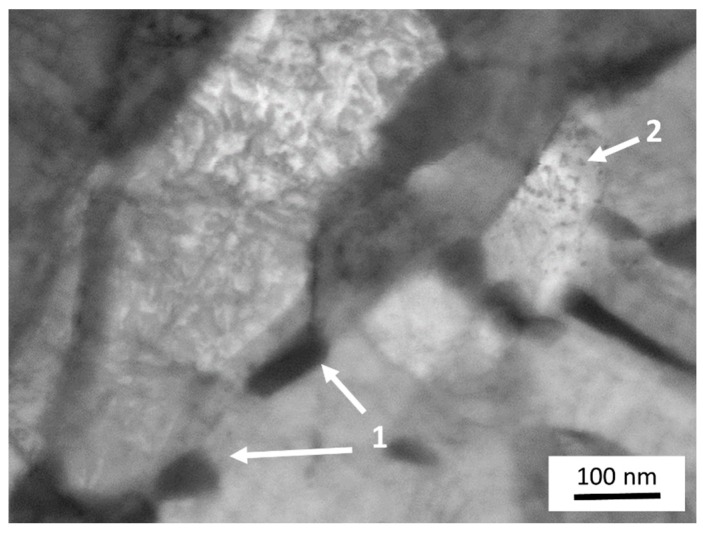
Scanning transmission electron microscopy STEM micrograph of a lamella prepared from the 1Cu-1Mn material which was quenched and tempered at 500 °C for 60 min. Individual martensite crystals appear as areas that differ in brightness. Large dark cementite particles are visible. There is one bright grain with clearly visible small precipitates within.

A thin lamella was prepared using FIB from the material 1Cu-1Mn which had been annealed at 500 °C for 60 min. A STEM micrograph taken at 30 kV is presented in Figure 6. Most of the martensite crystals do not offer good contrast or a sharp image of their internal structure. However, there is one grain which shows fine particles within, as well as faint lines connected to them. These can be interpreted as Cu precipitates pinning dislocations in the crystal of tempered martensite.

## 4. Discussion

Strengthening effect of Cu can be assessed from mechanical properties of experimental materials. Comparison of YS and UTS among materials subjected to the same thermal treatment revealed influence of alloying elements. Influence of Cu can be seen in content range 0–1–1.5%. Mn content was increased from 1% to 2% in Cu-alloyed materials to see its possible interference with the Cu-related strengthening.

Different strengthening mechanisms has to be taken into account. Grain boundary strengthening is the one present in all examined states—only quenched and also after tempering. Grains in studied materials are martensite crystals. Their maximal size is given by the size of the PAG. PAG size was nearly the same for all studied materials (Table 2). The martensite crystal size and morphology differs significantly within one PAG, because martensite crystals gradually fill its volume as the temperature drops from the martensite start to the martensite finish. No quantitative assessment of martensite crystals size was performed but microstructures were compared at the same magnifications (Figure 2). No visible difference was observed. Thus, there is no evidence for a significantly different contribution of grain boundary strengthening among studied materials. This assumption is valid for quenched state but also for the tempered states. The size of martensite crystals was not expected to change during the tempering, because the highest tempering temperature was 500 °C.

Solid solution strengthening can be assessed for the quenched state. C, Mn, and Cu were assumed as fully dissolved in a solid solution for the purpose of the models. Models for solid solution hardening were applied and increment ΔYS for all materials was computed based on the chemical composition from Table 1. The difference ΔYS is defined for certain material xCu-yMn simply as:

The solid solution strengthening increment of YS _C, Mn_ caused by the different content of C and Mn was considered as:

ΔYS _xCu-yMn_ = YS _xCu-yMn_ − YS _0Cu-1Mn_ (1)

(2) $$\Delta \mathrm{YS}\;{}_{C,\;\mathrm{Mn}}=1997\cdot \left(\Delta C\cdot \left(1+\frac{\Delta \mathrm{Mn}}{3.5}\right)\right)$$

where ΔC and ΔMn are differences in C and Mn content respectively (in wt.%) [20]. The solid solution strengthening increment of YS _Cu_ caused by the different content of Cu was considered for the martensitic matrix:

ΔYS _Cu_ = 17·(Cu_1_^1/2^ − Cu_2_^1/2^) (3)

where Cu_1_ and Cu_2_ are atomic concentrations of Cu in compared materials [21]. Overall model for ΔYS based on differences in C, Mn and Cu content was summed as

ΔYS _model_ = ΔYS _Cu_ + ΔYS _C, Mn_ (4)

**Figure 7:**
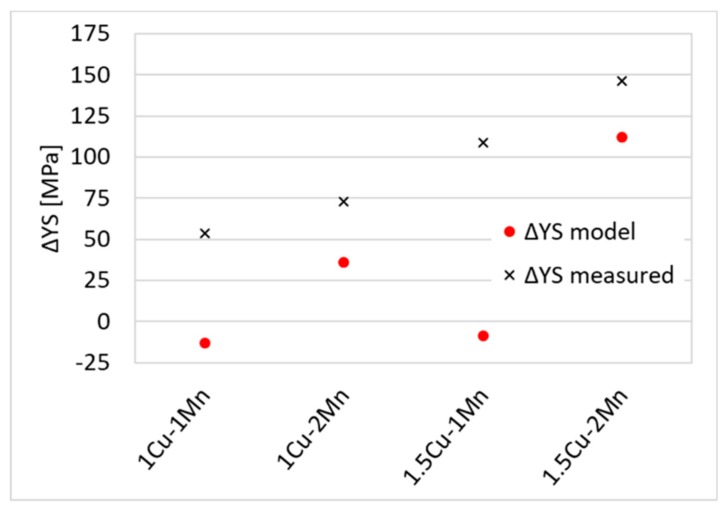
ΔYS for quenched state of different materials. YS _0Cu-1Mn_ is the zero-line.

Figure 7 shows that there is not a good agreement between the model of solid solution strengthening by C, Mn and Cu and the measured increase in YS. Cu contribution to the strengthening is apparently higher than model values. 

A possible reason for disagreement of measured and model data can be self-tempering of the martensite. Evidence for self-tempering can be seen in Figure 2b. The fine particles in some martensite crystals or in between them seemed to correspond to the reported cases of martensite self-tempering during quenching [22]. These fine particles were observed in martensite from all tested materials. Martensite crystals loose strength upon carbon release from a supersaturated solution [23,24]. The degree of self-tempering could influence significantly YS and UTS of martensite. Retained austenite content is another phenomena influencing the martensite YS. One of the mentioned phenomena has to be influenced by Cu and Mn content to cover the discrepancy of solid-solution model and the measured data. Influence of the Cu content contributes apparently much more than is predicted for the solid solution strengthening.

Tempering at the temperature 300 °C showed remarkable levelling of UTS and YS values of all materials in comparison with quenched state and higher tempering temperatures. Time-independent values of UTS and YS shows that temperature 300 °C was insufficient to trigger copper precipitation in the duration of 120 min; at least in extent which would influence the mechanical properties. The decrease of UTS can be therefore attributed to the carbon release from martensite. Solid solution strengthening was apparently not the main reason for differences in UTS and YS among the materials. The highest alloying did not generally lead to the highest values and vice versa. 

The tempering at 400 °C showed clearly the kinetic of the parallel processes—martensite tempering can be observed in case of material 0Cu-1Mn and influence of copper precipitation on it in case of Cu alloyed material. Differences between the materials after 15 min. tempering were comparable with values obtained from tempering at 300 °C. The difference of UTS and YS among the materials gradually increased with tempering prolongation from initial circa 60 MPa to 195 MPa. 

**Figure 8:**
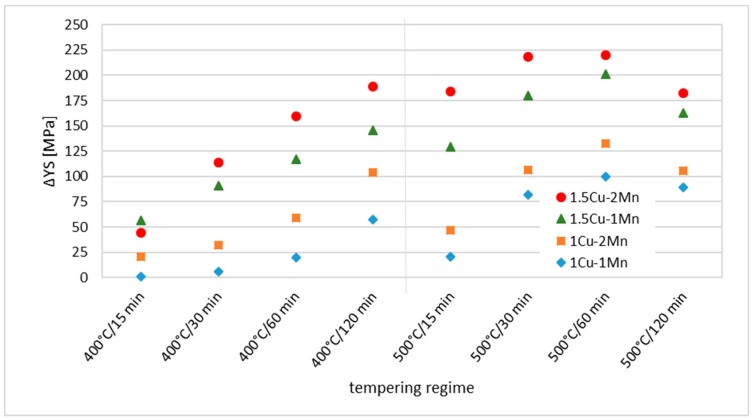
ΔYS for different materials in dependence of the tempering regime. YS _0Cu-1Mn_ is the zero-line.

The trend of ΔYS evolution with tempering time and the temperature is shown in Figure 8. Material 1Cu-1Mn did not exhibit significant strengthening for 15 and 30 min tempering at 400 °C. Gradual strengthening can be observed at longer annealing times 60 and 120 min. Material 1Cu-2Mn followed the trend of the 1Cu-1Mn with an offset to higher YS. Material 1.5Cu-1Mn showed strengthening from the 15 min. tempering and the ΔYS _1.5Cu-1Mn_ rose steadily to the tempering time 120 min. Addition of Mn to the concentration 2 wt.% retained behaviour of steady increase in ΔYS, but the growth rate of ΔYS was noticeably different. Tempering time 120 min was not enough to determine maximum strengthening of material because longer times are required to observe the descent phase of ΔYS with time. However, faster onset of strengthening with increasing Cu content was proved. Mn content in range 1 wt.% and 2 wt.% did not affect the kinetic of Cu precipitation strengthening. 

Tempering at 500 °C showed maximum strengthening achievable at that temperature. All materials with Cu exhibited a peak in strengthening at 60 min tempering duration. The decrease of ΔYS from 60 to 120 min. of tempering is presumably caused by coarsening of the Cu precipitates. The kinetics of precipitation strengthening was identical for all materials alloyed by Cu. Different Cu content resulted only in an offset in ΔYS values. 

The observed strengthening was determined as a difference between YS of Cu-containing steel and reference 0Cu-1Mn material. The highest strengthening was achieved after annealing at 500 °C for 60 min for materials 1Cu-1Mn and 1.5Cu-1Mn: 100 MPa and 200 MPa, respectively. The magnitude of strengthening can be compared with previously published studies. 300 MPa YS increase was reported for 1.4% Cu addition in almost pure iron after annealing at 500 °C for dwell in range from 2 to 8 h [7]. Very similar increase in UTS (290 MPa) was caused by addition of 1.5% Cu after annealing at 550 °C for 1 h [25]. Both studies performed analogous heat treatment by austenitization, fast cooling and annealing. However, martensitic structure was not formed due to low level of carbon (below 0.005%) and absence of other alloying elements. The strengthening was observed without concurrent martensite tempering and was larger by circa 50% than the maximal strengthening observed in the experiment. A possible explanation can be in different effectivity of precipitation strengthening in coarse-grained ferritic structure and fine-grained tempered martensite. Martensite crystals were usually thinner than 1 micrometer and they were strengthened not only by grain boundaries, but also by cementite particles precipitated in their interior. Dislocations movement is impaired in tempered martensite tremendously in comparison with large ferrite grain. Addition of precipitates of given size and density can manifest in ferrite with much lower concurrence of other strengthening mechanisms. 

Cu induced strengthening was examined also for low-carbon Ti-B alloyed steel [9]. Samples underwent controlled rolling with final rolling temperature 750 °C and subsequent cooling in air. Reported strengthening was 100 MPa for YS and 200 MPa for UTS caused by 1.6% Cu addition.

Increase of Mn content from 1 wt.% to 2 wt.% also caused an increase in ΔYS values for both Cu concentrations. Interesting behaviour can be observed especially in the case of 1.5Cu materials. The difference ΔYS caused by different Mn content is significantly lower in case of lower tempering (300 °C or 400 °C/15 min.) or tempering above optimal strengthening effect (500 °C/120 min). ΔYS values for material 1.5Cu-1Mn and 1.5Cu-2Mn in Figure 5 are further apart in case of tempering 400 °C/60 min., 400 °C/120 min, 500 °C/15 min. and 500 °C/30 min. This fact can indicate that Cu precipitation and Mn alloying act in synergy in case of steel strengthening. This effect is not clearly observable in the case of 1Cu materials.

Influence of different Mn content was examined by Maruyama et al [26]. Mechanical properties were studied by hardness measurement. Cu precipitation hardening was studied after 30 min. annealing at temperatures between 500 °C and 750 °C for Mn content 0.02% and 1.9%. It was found that Mn caused decrease of strengthening increment in over-aged region. This is consistent with the mechanical properties in the over-aged samples annealed at 500 °C for 2 h. Increased Mn content lead to sharper decrease in YS.

## 5. Conclusions

Quenching and tempering of 0.2% C steels with different Cu contents (0.1%, 1.0% and 1.5%) and different Mn contents (1% and 2%) was performed. The experimental steels exhibited different tensile strengths and yield stress levels in the as-quenched state. The differences were larger than those expected solely on the basis of solid solution strengthening of martensite, particularly with regard to different Cu levels. Explanation of this phenomenon requires a detailed examination of as-quenched materials, in terms of the martensite crystals size and morphology, retained austenite content and the extent of martensite self-tempering.

Higher tempering temperatures, 400 °C and 500 °C, caused tempering of martensite, as well as copper precipitation, which significantly strengthened the tempered martensite structure. The strengthening was time-dependent. A peak in strengthening was observed at 500 °C and 60 min.

Possible strengthening synergy between Cu and Mn was observed in steels with the highest Cu content (1.5%). The increase in the Mn content from 1% to 2% resulted in higher UTS and YS. This increase corresponded with the presumed effects of solid solution strengthening for low- and high-tempered states in the experiment. However, a higher Mn level caused higher strengthening in the mid-tempered states in the experimental program. In that particular case, the contribution from Mn cannot be explained as plain solid solution strengthening.

## Figures and Tables

**Table 1:** Chemical compositions of experimental heats in wt.%.

Material	C	Cu	Mn	Si	Ti	B	N
0Cu-1Mn	0.22	0.12	0.98	0.07	0.022	0.0014	0.0056
1Cu-1Mn	0.21	1.08	0.98	0.08	0.025	0.0013	0.0063
1Cu-2Mn	0.19	0.98	1.91	0.08	0.025	0.0013	0.0053
1.5Cu-1Mn	0.21	1.49	0.99	0.10	0.022	0.0013	0.0054
1.5Cu-2Mn	0.21	1.49	2.00	0.08	0.023	0.0015	0.0057

**Table 2:** Characteristics of the as-quenched state. UTS—ultimate tensile strength, YS—yield stress, Ag—uniform plastic elongation, A_5_—total plastic elongation, HV10—Vickers hardness, d—average PAG diameter.

Material	UTS (MPa)	YS (MPa)	Ag (%)	A_5_ (%)	HV10	d (µm)
0Cu-1Mn	1447	1057	3.6	13.9	478	9.1
1Cu-1Mn	1495	1110	3.1	11.2	475	10.3
1Cu-2Mn	1472	1129	3.1	11.3	452	9.7
1.5Cu-1Mn	1520	1166	1.9	12.9	470	10.1
1.5Cu-2Mn	1594	1203	2.7	13.5	482	9.2
